# Underwater image restoration with Haar wavelet transform and ensemble of triple correction algorithms using Bootstrap aggregation and random forests

**DOI:** 10.1038/s41598-022-11422-2

**Published:** 2022-05-27

**Authors:** Vahid Rowghanian

**Affiliations:** Independent Author, Ahvaz, Iran

**Keywords:** Physical oceanography, Computer science

## Abstract

This paper presents both a new strategy for traditional underwater image restoration using Haar wavelet transform as well as a new learned model that generates an ensemble of triple correction algorithm labels based on histogram quadrants’ cumulative distribution feature instead of generating pixel intensities. The Haar wavelet transform is our tentative dynamic stretching plan that is applied on the input image and its contrast stretched image to generate the degraded wavelet coefficients which are blended using Gaussian pyramid of the saliency weights to restore the original image. The ensemble of triple corrections exerts three color correction algorithms sequentially on the degraded image for restoration. The ensemble of algorithms entails the superposition effect of the red channel mean shifting, global RGB adaptation, global luminance adaptation, global saturation adaptation, luminance stretching, saturation stretching, contrast stretching, adaptive Gamma correction for red spectrum, even to odd middle intensity transference using look-up table, green to red spectrum transference using histogram equalization, local brightening, Dark Channel Prior, fusion restoration, and our Haar wavelet transform restoration. The source is available at https://github.com/vahidr213/Underwater-Image-Restoration-And-Enhancement-Collection.

## Introduction

Various researchers have recently visualized several mathematical methods and thoughts to define an effective solution for color losses in underwater images and limitations by the extent to which several literatures in the machine vision science have approached the underwater image restoration problem from different perspectives. On the other hand, underwater image restoration is by no means trivial. It is actually as challenging as most in machine vision and image processing.

The primary factor in underwater image degradation is the high attenuation of the radiated light from the surface of the objects^1^. An early and famous single image haze removal for outdoor images named Dark Channel Prior (DCP) introduced the assumption of darkness in at least one of the RGB channels in any non-sky patch of the image. Underwater Dark Channel Prior (UDCP)^2^ is derived from the DCP^3^ for underwater images assuming the red channel as the dark channel, hence using green and blue channels to estimate the medium transmission matrix.

From the application point of view, 3D reconstruction of seafloor encounters objects in images that require automatic true color recovery since the effects can limit the ability to assess the changes happened to underwater organisms such as those living in benthic zone^4^.

The Sea-thru method^5^ examined the dark channel prior in detail via finding the optimized medium transmission matrix by estimating the range-dependent attenuation coefficient using local space average color. An alternative to dark channel prior named red channel prior^6^ is a rearrangement of the original DCP equation as well as refining the estimated transmission map with guided filter instead of matting technique. Recently, the transmission map of the DCP method for the red channel have been estimated^7^ by applying adaptive non-convex and non-smooth variation to a rough initial transmission map and then using this optimized transmission map to obtain red global background light via thermal exchange optimization, then the refined red channel is recombined with green and blue channels to form the restored image. As mentioned before, a complex image formation model is proposed^1^ that has drawn attention to be utilized in^8^ for depth and background light estimation. In^9^, an attempt toward the estimation of background light and the transmission map is performed by using the feature priors of flatness, hue, and brightness.

Despite the prolific works as yet presented DCP and UDCP approaches, none have comprehensively sufficient positive effect on the underwater image restoration problem since the dark channel prior formation model is regulated for the atmospheric haze and due to being a weak function of wavelength, it's inadequate to be rendered for light propagation in harsh oceanic environments^1^.

Other strategies such as multi-scale fusion has drawn much attention in recent years. The method proposed by Ancuti et al.^10^ has been built by fusing four weights (namely, exposedness, Laplacian of luminance, locally averaged luminance and saliency) extracted from both a contrast stretched image as well as an equalized luminance image to determine how each pixel should be modified.

Inspired by UDCP and multi-scale fusion, an algorithm is proposed^11^ so that the medium transmission map produced by UDCP is decomposed into saliency and Laplacian weights. These weights are joined by multi-scale fusion to produce a refined medium transmission map which is used in DCP image formation model to restore the original image. However, fusion methods mentioned above produce equalized distributions in RGB spectrums that can cause over-enhancement or under-enhancement at some regions of image where the distribution is yet to remain untouched or shall have carried to the farthest ends of the intensity distribution. Moreover, saliency weights in both methods largely take up the principal structure of the weightings in contrast to other weights which have negligible effect.

Over the past decades, convolutional neural networks (CNN) have extended in many visual recognition fields. An encoding–decoding convolutional neural network^12^ is used to restore underwater images where the encoding and decoding levels are visualized by convolution and deconvolution operations respectively. The inner structure of the convolutional part is similar to the popular Alexnet network. They have accelerated the training process of the CNN as well as including low-level features by using skip connection between the encoding and decoding stages. The skip connection has been used since few convolutions extract some important features, but it can smooth the image and destruct the edges if over applied especially in underwater images.

Neural networks are powerful and capable of constructing various architectures. Some of published works in the past few years, are domain adversarial learning^13^, generative adversarial network (GAN)^14^, underwater convolutional NN^15^, multi-scale dense block GAN^16^ using batch normalization^17^, underwater joint residual learning CNN^18^, underwater image restoration network^19^, WaterGAN^20^, MCycleGAN^21^, underwater image enhancement with stacked conditional GAN^22^.

The above mentioned networks still reflect a considerable rate of failure due to lack of handling the saturation and contrast problems. In^23^, an end-to-end CNN with three blocks named RGB (for basic operations), HSV (saturation and luminance adjustment) and attention (stage for quality enhancement) is proposed where the final restored image is produced by a weighted sum between the RGB block output and the attention block’s RGB component as well as a weighted sum between HSV block output and the attention block’s HSV component. The input of the attention block is a concatenation of the raw image and the images of other two blocks (RGB block and HSV block). As a good survey, some of above mentioned methods as well as other methods are recapitulated in^24^.

The following literature explains the proposed method, the inputs, the structure of the multi-scale fusion restoration and 2-D Haar wavelet transform restoration, the structure of the Ensemble of Triple Correction Algorithms (ETCA) and finally the experimental results and comparison with other state of the art algorithms.

## Proposed method

**Figure 1:**
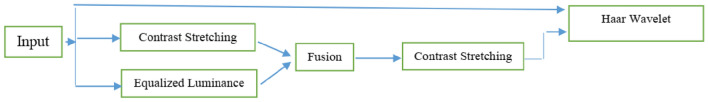
The flowchart of the multi-scale fusion ^10^ and Haar wavelet transform for underwater image restoration.

In this paper, our contribution to the problem of underwater image restoration includes a traditional method as well as a learned model using new features. The traditional methods solve portions of the whole problem quite topnotch. We used an alternative single image-based solution through blending the Haar wavelet coefficients of the raw image with its contrast stretched version (static tolerance) to convey a sense of dynamic contrast stretching. Our approach is applied after the multi-scale fusion restoration^10^ to further improve the quality of the restored image. The flowchart of this approach is shown in Fig. 1.

On the other hand, a comprehensive method for random patterns requires trained models. In this paper, we use the histogram quadrants’ cumulative distribution as a new feature to characterize each image instead of using pixel intensity. The features are then learned using Bootstrap Aggregation and Random Forests. We have trained three models separately. The responses of models are in the form of a numeric label instead of pixel intensities. Each model generates a numeric label corresponding to a special color correction algorithm that should be applied sequentially to restore the original image.

### Input images

The input images of fusion process can generally be different because the fusion is based on combining multiple sources to preserve the significant features of them. In this regard, the contrast stretching is one of the most popular initial color correction tools as yet has overcome significant initial white balancing. Numerous white balancing methods have been suggested but none of them are always experimentally appropriate in underwater scenes. The Gray-World approach of Bachsbaum et al.^25^ is a mediocre example of white balancing methods, yet creates color deviation by introducing reddish artifacts where the appearance is overall blue. The following method is used to adjust white balance initially which is simple and efficient and creates less red artifacts. At first, a three-element vector is defined as below:

1 $$ratio = K\left[ {\frac{{I_{\max }^{R} }}{{\overline{I}^{R} }}, \frac{{I_{\max }^{G} }}{{\overline{I}^{G} }}, \frac{{I_{\max }^{B} }}{{\overline{I}^{B} }}} \right]$$

where *K* is a constant (e.g. 0.005) to make a vector of probability ratios in the interval [0 1]. The three cumulative probabilities presented in *ratio* and their complements in (*1-ratio)* determine the percentage of the data that should be saturated to the lowest and highest values in each of the three RGB channels respectively.

### Haar wavelet inputs

The Haar wavelet transform is fed with the raw input image as well as a %0.5 contrast stretched version of the raw image through the method described above. The input image for Haar transform is resized into 640 × 640 in order to have the Haar pyramid dimensions as equal as the Gaussian pyramid of the blending weights (W_1_, W_2_) as well as considering a power of 2 dimension to facilitate the wavelet transform. The input image can also be resized to 1024 × 1024 to increase the accuracy.

#### Multi-scale fusion input

The first image for the pyramid of multi-scale fusion^10^ is the contrast stretched version of the input image explained above and the second one is an equalized luminance image provided by adaptive histogram equalization^26^.

### Weights of multi-scale fusion

The choice of correct weights reduces computations and artifacts. There are four weights in^10^ that are explained below:

#### Laplacian contrast weight (W_L_)

Laplacian contrast weight is the absolute value of the Laplacian filter applied on the luminance component of the image.

#### Local contrast weight (W_LC_)

Local contrast weight is an additional contrast measure to recover the contrast in the regions where the Laplacian contrast weight (W_L_) is not sufficient (e.g. ramp and flat regions). It improves the transitions between dark and bright regions. The *(W*_*LC*_*)* is computed as the square of the difference between the luminance and its locally averaged luminance (e.g. 5 × 5, [1,4,6,4,1]/16 filter plus cutting off values above $\pi /2.75$ to $\pi /2.75$).

2 $$W_{LC} \left( {x,y} \right) = \left( {L - \overline{L}} \right)^{2}$$

#### Saliency weights (W_S_)

Saliency weights emphasize on the pixels standing farthest relative to the mean of the reddish, bluish and luminance components collectively. In other words, the salient objects in a scene are biologically recognized and similarly mathematically defined as yet taken up a region of center-surround contrast. The saliency algorithm of Achanta et al.^27^ is used which is defined in Lab color space by:

3 $$W_{S} \left( {x,y} \right) = \left( {L\left( {x,y} \right) - \overline{L}} \right)^{2} + \left( {a\left( {x,y} \right) - \overline{a}} \right)^{2} + \left( {b\left( {x,y} \right) - \overline{b}} \right)^{2}$$

#### Exposedness weights (W_E_)

Exposedness weights are a rough estimation of how much a pixel is exposed to light. This weight is defined by a Gaussian profile resembling (^) sign with mean value 0.5 and standard deviation 0.25. Therefore, pixels close to the average value are exaggerated to produce better results when combined with saliency weights. Exposedness weight (W_E_) is defined as:

4 $$W_{E} \left( {x,y} \right) = {\text{exp}}\left( { - \frac{{\left( {I\left( {x,y} \right) - 0.5} \right)^{2} }}{{2\sigma^{2} }}} \right)$$

The final weights W_1_ and W_2_ combine the above mentioned four weights and have the same size as input image (like all four weights). The final weights determine the amount of modifications that each pixel will receive after the fusion applied. The final weights W_1_ and W_2_ are computed as below:

5 $$W_{1} = \frac{{W_{L1} + W_{LC1} + W_{S1} + W_{E1} }}{{W_{L1} + W_{LC1} + W_{S1} + W_{E1} + W_{L2} + W_{LC2} + W_{S2} + W_{E2} }}$$

6 $$W_{2} = \frac{{W_{L2} + W_{LC2} + W_{S2} + W_{E2} }}{{W_{L1} + W_{LC1} + W_{S1} + W_{E1} + W_{L2} + W_{LC2} + W_{S2} + W_{E2} }}$$

### Haar wavelet coefficient blending

**Figure 2:**
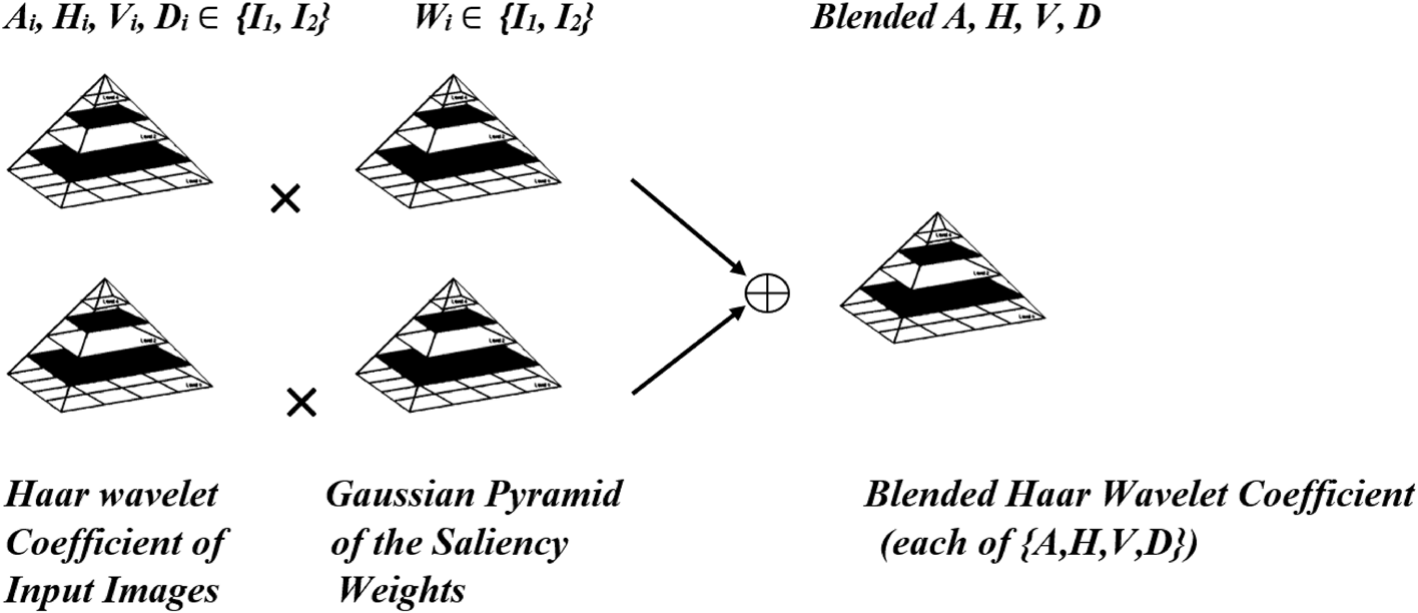
The block diagram for illustrating the Haar wavelet coefficient refinement.

Wavelet and inverse wavelet transformations have been designed as an analyzing and synthesizing tool for signals and images. The Haar wavelet transform has a good potential to decompose images into Approximation, Horizontal, Vertical and Diagonal components. Thus, it is suitable for multi-scale blending of coefficients to convey a sense of tentative dynamic stretching. A %0.5 contrast stretched image is derived from the initial degraded image (I_2_). Therefore, it can generate slightly different type of wavelet coefficients which still carry degradation information. In this paper, we propose to blend the degraded Haar wavelet coefficients {A, H, V, D} of the two images (I_1_, I_2_) using the Gaussian pyramid of the saliency weights ($W_{1} ,W_{2}$) to achieve new coefficients (A_new_, H_new_, V_new_, D_new_). Figure 2 demonstrates the structure of the coefficient blending.

In the blending process, the saliency weights are used solely since the saliency weights take up the principal structure among the four weights described above. The refinement or blending weights ($W_{1} ,W_{2}$) are computed according to the following equations:

7 $$W_{1} = \frac{{W_{S1} }}{{W_{S1} + W_{S2} }}$$

8 $$W_{2} = \frac{{W_{S2} }}{{W_{S1} + W_{S2} }}$$

The blending of the degraded Haar wavelet coefficients are performed according to the following equations:

9 $$H_{C\,new}^{l} \left( {x,y} \right) = \mathop \sum \limits_{k = 1}^{K} G^{l} \left\{ {W^{k} \left( {x,y} \right)} \right\} H^{l} \left\{ {I_{c}^{k} \left( {x,y} \right)} \right\}$$

10 $$V_{C,\,new}^{l} \left( {x,y} \right) = \mathop \sum \limits_{k = 1}^{K} G^{l} \left\{ {W^{k} \left( {x,y} \right)} \right\} V^{l} \left\{ {I_{c}^{k} \left( {x,y} \right)} \right\}$$

11 $$D_{C,\,new}^{l} \left( {x,y} \right) = \mathop \sum \limits_{k = 1}^{K} G^{l} \left\{ {W^{k} \left( {x,y} \right)} \right\} D^{l} \left\{ {I_{c}^{k} \left( {x,y} \right)} \right\}$$

12 $$A_{new} = \mathop \sum \limits_{k = 1}^{K} G^{l} \left\{ {W^{k} \left( {x,y} \right)} \right\}A^{k}$$

where $H^{l} \left\{ {I_{c}^{k} \left( {x,y} \right)} \right\}$, $V^{l} \left\{ {I_{c}^{k} \left( {x,y} \right)} \right\}$, $D^{l} \left\{ {I_{c}^{k} \left( {x,y} \right)} \right\}$ are the horizontal, vertical and diagonal coefficients of *kth* image for the channel $c \in \left\{ {R,G,B} \right\}$ at level *l*. *A*_*k*_ is the approximation coefficient matrix for image *k*.

The inverse Haar transform is then applied on the refined coefficients (A_new_, H_new_, V_new_, D_new_) to restore the original image.

The Haar wavelet restoration is able to restore underwater images independently. Nevertheless, our experiments shown the overall improvement of efficiency through using the Haar wavelet restoration method as a complementary algorithm. Thus, we applied the Haar wavelet restoration method on the restored image produced by multi-scale fusion^10^. Figure 1 shows the flowchart of our Haar wavelet restoration and multi-scale fusion^10^ structure. The overall algorithm shown a record breaking result representing an average mean square error (MSE) better than almost all trained models and traditional methods except for Dive + algorithm.

### Multi scale fusion

The fusion is represented by a weighted sum of images at every location (x,y):

13 $$R\left( {x,y} \right) = \mathop \sum \limits_{k = 1}^{K} \overline{W}^{k} \left( {x,y} \right)I^{k} \left( {x,y} \right)$$

where $I^{k}$ (*MxN*) is the input image (*k* = *1,2,…,K*) with *M* rows and *N* columns, $\overline{W}^{k}$ (*MxN*) is the normalized weights (*k* = *1,2,…,K*) ($\sum \overline{W}^{k} = 1$) and *R* is the restored image. $I^{k}$ can generally be any color corrected form of the raw input image.

Due to halos and artifacts that are introduced in *R(x,y)* by directly applying equation above, both weights and input images are decomposed into a multi-scale pyramid defined below:

14 $$R^{l} \left( {x,y} \right) = \mathop \sum \limits_{k = 1}^{K} \left\{ {\overline{W}^{k} \left( {x,y} \right)} \right\}^{l} \left\{ {I^{k} \left( {x,y} \right)} \right\}^{l}$$

where *l* represents the number of pyramid levels. Each scale is derived by down sampling the previous level. The initial level is the original image.

To preserve the important and desired information, an operation such as Gaussian filtering or Laplacian filtering can be applied before down sampling. In this regard, we decomposed weights into a Gaussian pyramid and decomposed images into a Laplacian pyramid. Therefore, the equation above can be rewritten by:

15 $$R^{l} \left( {x,y} \right) = \mathop \sum \limits_{k = 1}^{K} G^{l} \left\{ {\overline{W}^{k} \left( {x,y} \right)} \right\}L^{l} \left\{ {I^{k} \left( {x,y} \right)} \right\}$$

where $L^{l} \left\{ {I^{k} \left( {x,y} \right)} \right\}$ is the Laplacian of the *kth* image at level *l*, and $G^{l} \left\{ {\overline{W}^{k} \left( {x,y} \right)} \right\}$ is the Gaussian of the *kth* weight at level *l*. The Gaussian pyramid is built by Gaussian filtering (or 5 × 5, [1,4,6,4,1]/16) and down sampling the image repetitively until we reach to the maximum level, considering the first layer to be the same size but only filtered version of the input image.

The Laplacian pyramid is created by building a pyramid of down sampled images at first, then calculating the difference of the lower level image from the resized (enlarged) upper level image (using bi-cubic interpolation). The first level for both the Gaussian pyramid and the Laplacian pyramid is as the same size as the input image to increase the accuracy.

## Ensemble of triple corrections

Traditional methods present a global solution (or limited joint solutions) for a typical prior assumption such as homogeneous lighting along the line of sight, unbalanced color spectrum in RGB channels, saturated colors, low contrast and brightness, blurriness, and especially degradation based on underwater image formation. On the other hand, the comprehensiveness of a specific traditional method solving different patterns of underwater degradation is as yet undecided.

**Figure 3:**
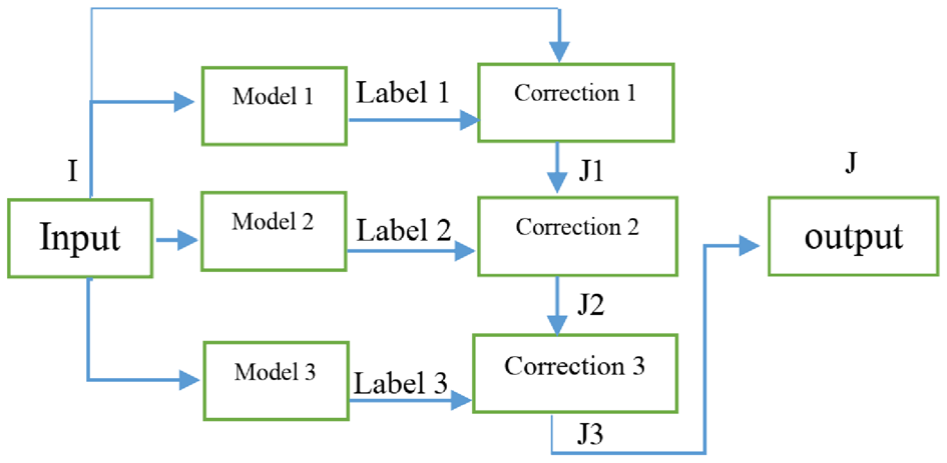
The block diagram illustrating the flow chart of the Ensemble of Triple Correction Algorithms. The three models generate three numeric labels for three stages (Correction 1, Correction 2, and Correction 3) to apply the corresponding color correction operation on the raw image.

**Figure 4:**
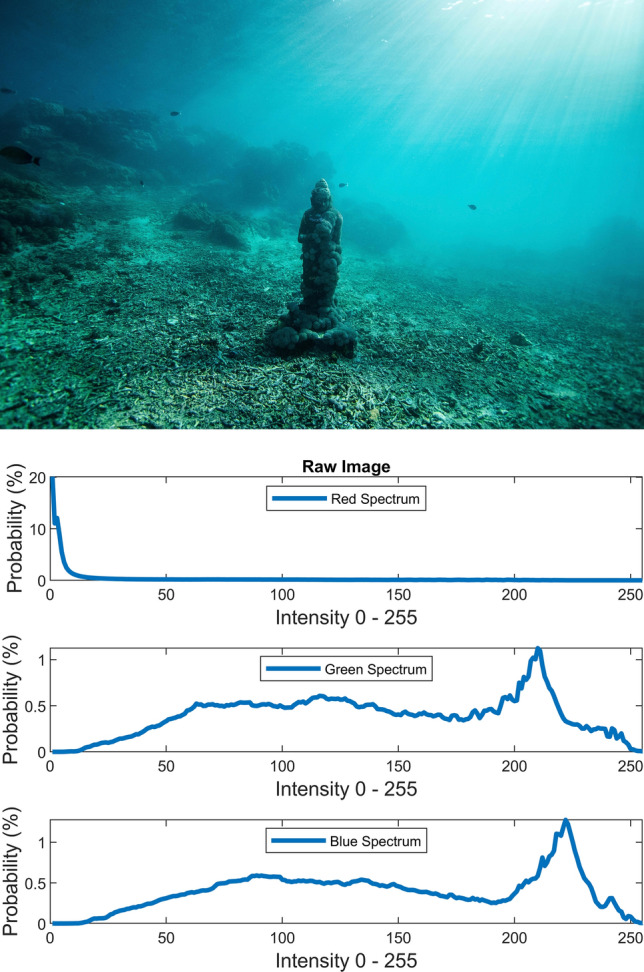
The raw image and the RGB histograms (from unsplash.com).

**Figure 5:**
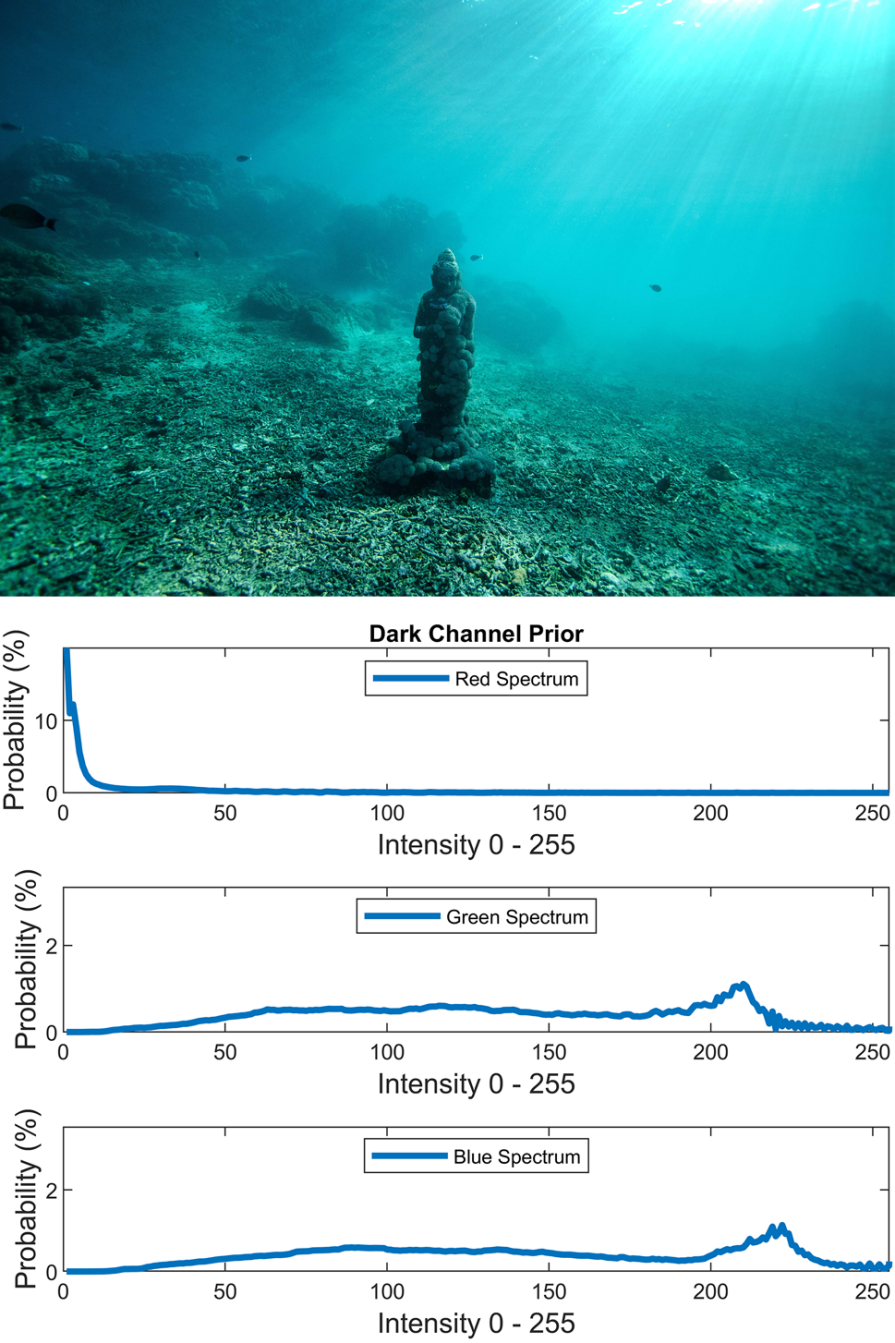
Dark Channel Prior.

**Figure 6:**
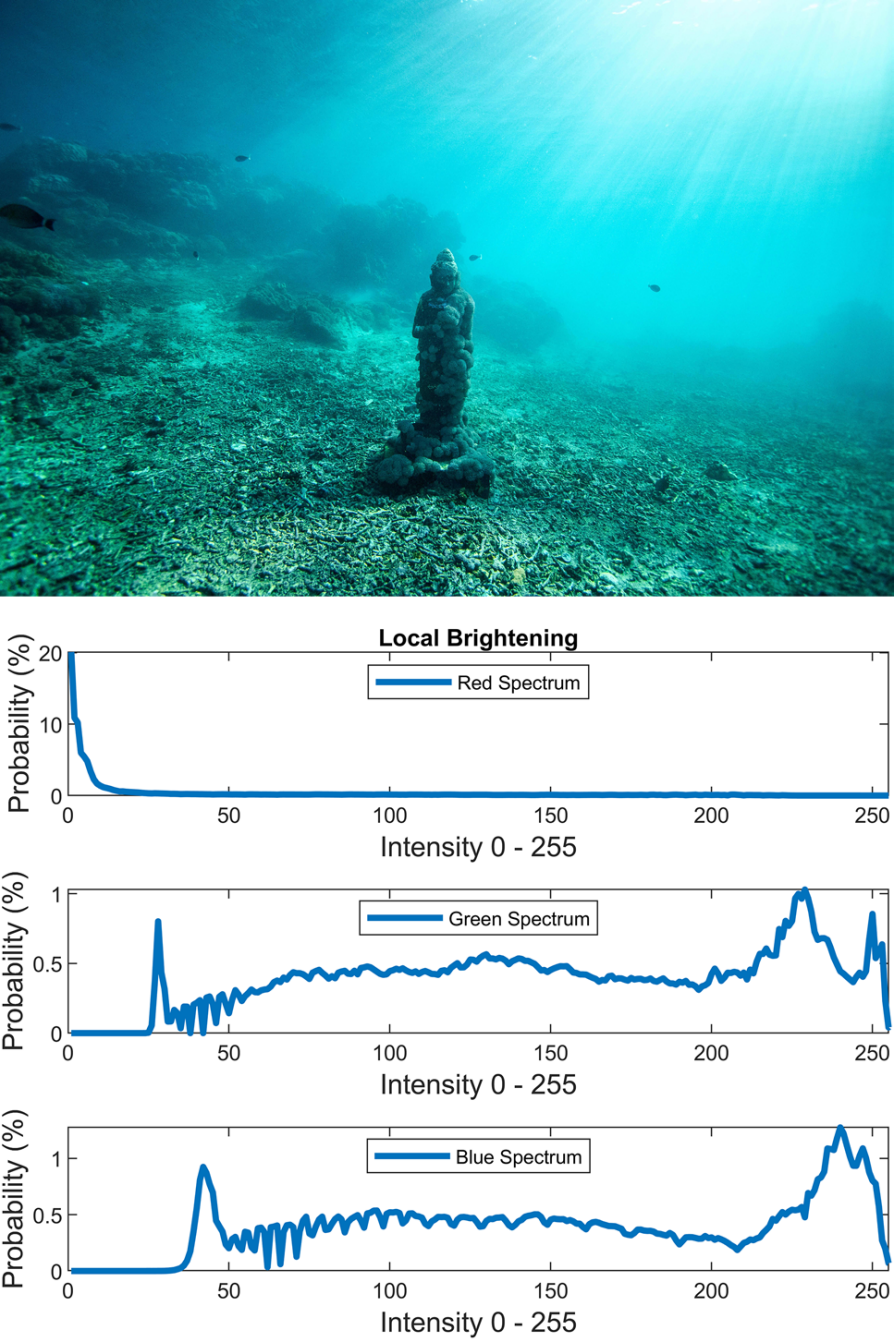
Local brightening.

There are several conventional color correction techniques aimed at increasing the visual quality of an image. These techniques include stretching, global adaptation, sharpening, unsharp masking, histogram equalization and many others. In this paper, we propose to predict and choose the best three combinations from an ensemble of conventional color correction techniques as well as our current proposed methods to be applied on a single image according to the features extracted from the image. In this paper, we have also proposed new features to be learned which are explained in detail in the feature extraction section. Three models are trained with these extracted features. The training method is Bootstrap Aggregation and Random Forests. Each model is responsible for generating a numeric label for its corresponding stage that indicates a specific color correction method among the fifteen available methods. It should be noted that the triple corrections are applied sequentially as the block diagram of the proposed method ETCA shows in Fig. 3. The ensemble of color correction methods entails the superposition effect of the red channel mean shift and sharpening, global RGB adaptation, global luminance adaptation, global saturation adaptation, luminance stretching, saturation stretching, contrast stretching, adaptive Gamma correction for red spectrum, our even to odd middle intensity transference using look-up table, green to red spectrum transference using histogram equalization, local brightening, DCP^3^, fusion restoration^10^, and our Haar wavelet transform restoration. For comparison purposes, Fig. 4 is used for all the subjective visualizations. Figures 5, 6 illustrate the visual and spectral effects of DCP and local brightening methods applied on Fig. 4.

The total number of the available methods including no action is equal to fifteen. It should be noted that the null operation leaves the image intact. This was necessary due to existence of some fully enhanced images in the UIEB dataset which had a negligible difference with their reference image (negligible mean square error). Other color correction algorithms are assigned a special numeric label identifying the corresponding operation. The color correction methods are described in the feature extraction section in detail.

### Features extraction

The feature extraction plays a key role in the quality of the deep learning-based underwater enhancement methods. As far as we know, most of the recent deep learning models have used the RGB values as their features. A majority of the state of the art underwater deep learning methods map pixel to pixel or pixel to difference. Pixel to pixel mapping mutually produces an output pixel in response to an input pixel. Pixel to difference mapping produces a positive or negative amount in the output to be added to or subtracted from the input pixel intensity. However, the possibility of generating multiple RGB combinations in the outputs of these networks still remain unsolved and will fall behind the generalization of the such conventional deep learning-based underwater restoration methods.

In this regard, we propose a probability based solution to be an alternative feature for pixel intensity. We have used cumulative distribution function (*CDF*) in 4 closed intervals or regions of the probability distribution function (*PDF*) of the input image. Therefore, each quadrant of the image’s *PDF* is assigned with a *CDF* scalar. Since 8-bit RGB images have three channels, a total of twelve scalars are extracted for each input image using equations below.

16 $$S_{1}^{c} = f^{c} \left( {I \le {\Omega }_{1} } \right)$$

17 $$S_{2}^{c} = f^{c} \left( {I \le {\Omega }_{2} } \right) - f^{c} \left( {I \le {\Omega }_{1} } \right)$$

18 $$S_{3}^{c} = f^{c} \left( {I \le {\Omega }_{3} } \right) - f^{c} \left( {I \le {\Omega }_{2} } \right)$$

19 $$S_{4}^{c} = f^{c} \left( {I \le {\Omega }_{4} } \right) - f^{c} \left( {I \le {\Omega }_{3} } \right)$$

where $S_{n}^{c}$ is the sum of probabilities in the *n* th interval, *f* is the probability distribution function or histogram, $c \in \left\{ {R,G,B} \right\}$, and ${\Omega }_{1} , \ldots ,{\Omega }_{4}$ are the maximum boundary of each quadrant which their numeric values are in the experimental results.

We have used these twelve *CDF*s as input features. The cumulative distribution (CDF) of each histogram quadrant decreases the number of training features significantly in contrast to huge training data used in above mentioned networks. The histogram quadrants also decrease the training time significantly.

The training process also needs the corresponding responds for which the input features have been extracted. In this work, the respond of the network is also not a pixel intensity. The respond to each input feature is an ensemble of optimized triple labels minimizing the cost function (in our case, the Mean Square Error between the reference image and the restored image). Each numeric label corresponds to a single special color correction method and each ensemble of triple labels has to be applied on the input image sequentially to restore the original image. Therefore, an optimization has been performed to generate the optimum triple correction methods (labels) for each input image.

A total of fifteen color correction methods used in evaluations. One of them is no action. Hereafter, other color correction methods are described in detail. It should be mentioned that the superiority of contrast stretching and pyramidal methods over other proposed methods with shallow positive effects is obviously not a strange phenomenon but the essence of beneficiary complementary approaches is exclusively indispensable for other aspects of visual improvements.

**Figure 7:**
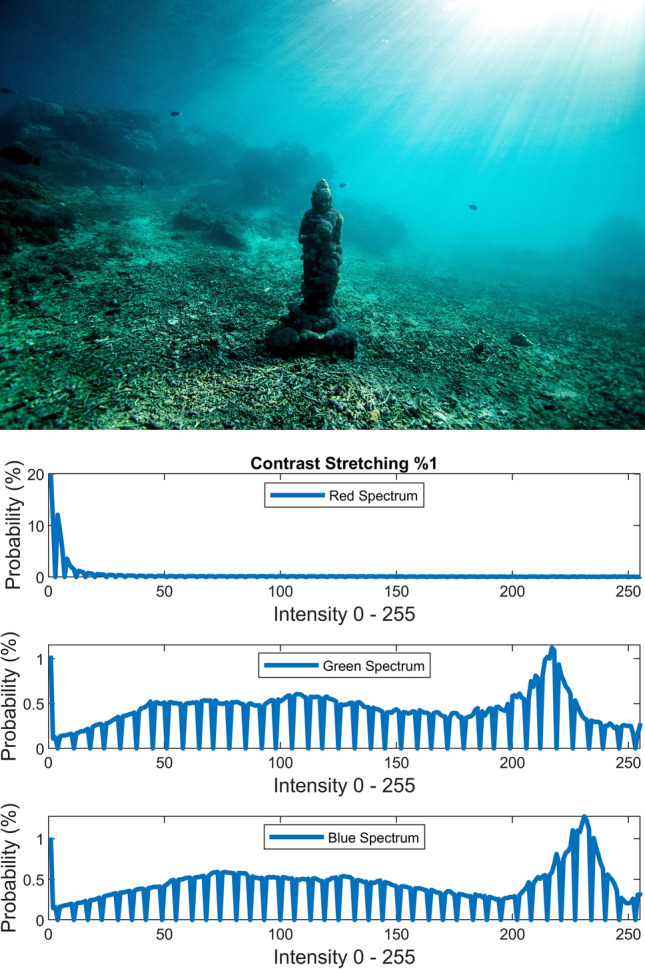
%1 Contrast stretching.

**Figure 8:**
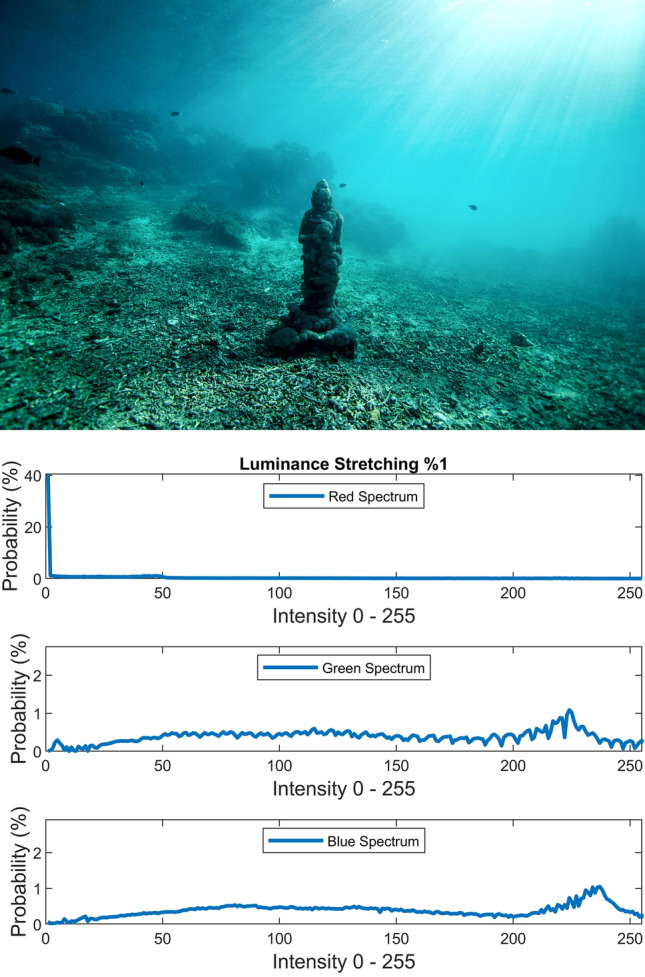
%1 Luminance stretching.

**Figure 9:**
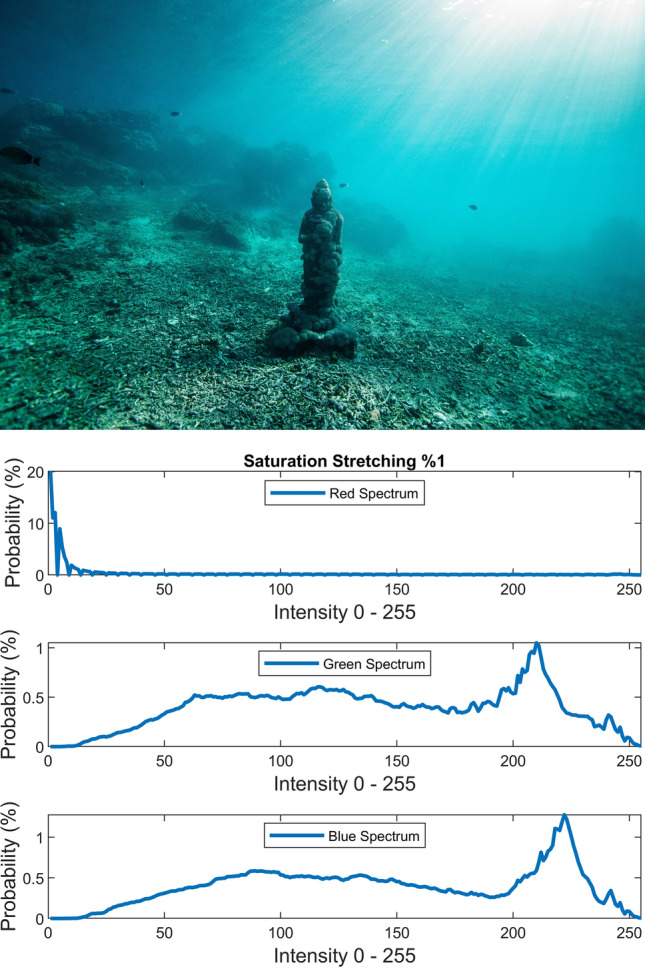
%1 Saturation stretching.
- The global RGB adaptation, global luminance adaptation and global saturation adaptation are performed using the logarithmic approximate function of human visual system (in early stages) according to the Weber-Fechner law^28^ and the retinex theory^29^. The global adaptation of an image I is expressed by:
  21 $$I_{g} \left( {x,y} \right) = \frac{{{\text{log}}\left( {1 + \frac{{I\left( {x,y} \right)}}{{\overline{I}}}} \right)}}{{{\text{log}}\left( {1 + \frac{{I_{max} }}{{\overline{I}}}} \right)}}$$
  where scalar $I_{max}$ is image maximum pixel intensity and the scalar $\overline{I}$ is the log-average of the image that enables the $I_{g}$ to adapt to the scene and is computed by equation below:
  22 $$\overline{I} = {\text{exp}}\left( {\frac{1}{MN} \sum \sum {\text{log}}\left( {I\left( {x,y} \right) + \delta } \right)} \right)$$
  where $I\left( {x,y} \right)$ is an $M \times N$ gray scale image and $\delta$ is small value to avoid singularity (e.g. $\delta = 10^{ - 3}$). As the log-average $\overline{I}$ increases to larger values, the global adaptation equation $I_{g}$ behaviors more in linear shape than logarithm shape. Therefore, darker scenes (i.e. contrast, saturation and luminance spectrums) are brightened more than brighter scenes through global adaptation. Figures 10, 11, 12 illustrate the visual and spectral effects of these global adaptation methods applied on Fig. 4.

- The static contrast stretching in RGB color space, static luminance stretching in Lab color space, and static saturation stretching in HSV color space are performed simply by using the following equation which saturates the bottom 1% and the top 1% of contrast, luminance, and saturation values (with proper *L* and *H*) to the lowest and highest values.
  20 $$J = \frac{I - L}{{H - L}}$$
  where *L* and *H* are the intensities corresponding to bottom 1% and top 1% of the image *I*. Figures 7, 8, 9 illustrate the visual and spectral effects of these stretching methods applied on Fig. 4.

**Figure 10:**
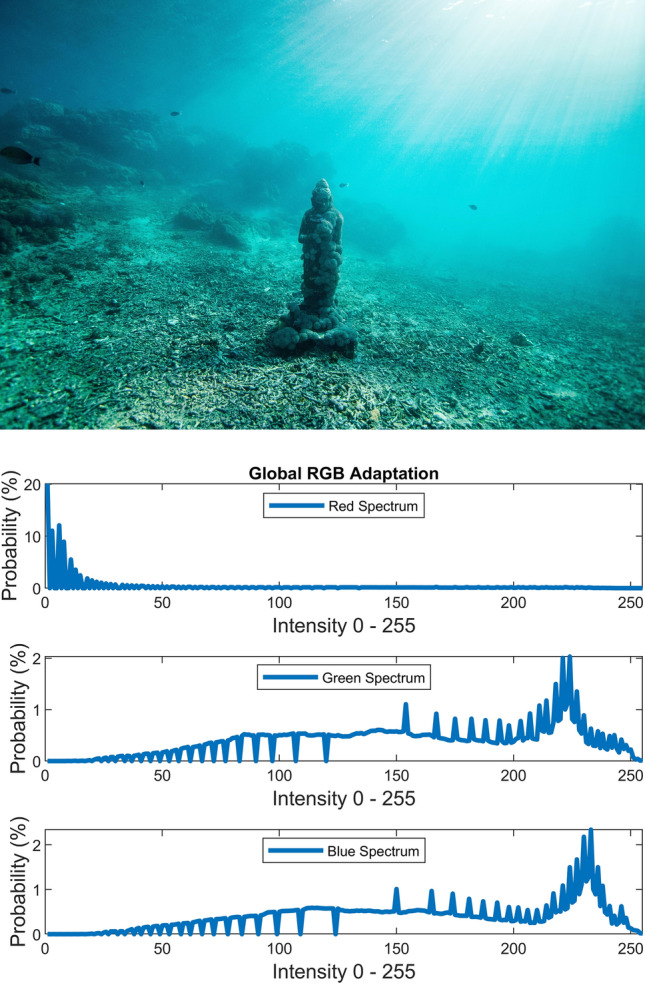
Global RGB adaptation.

**Figure 11:**
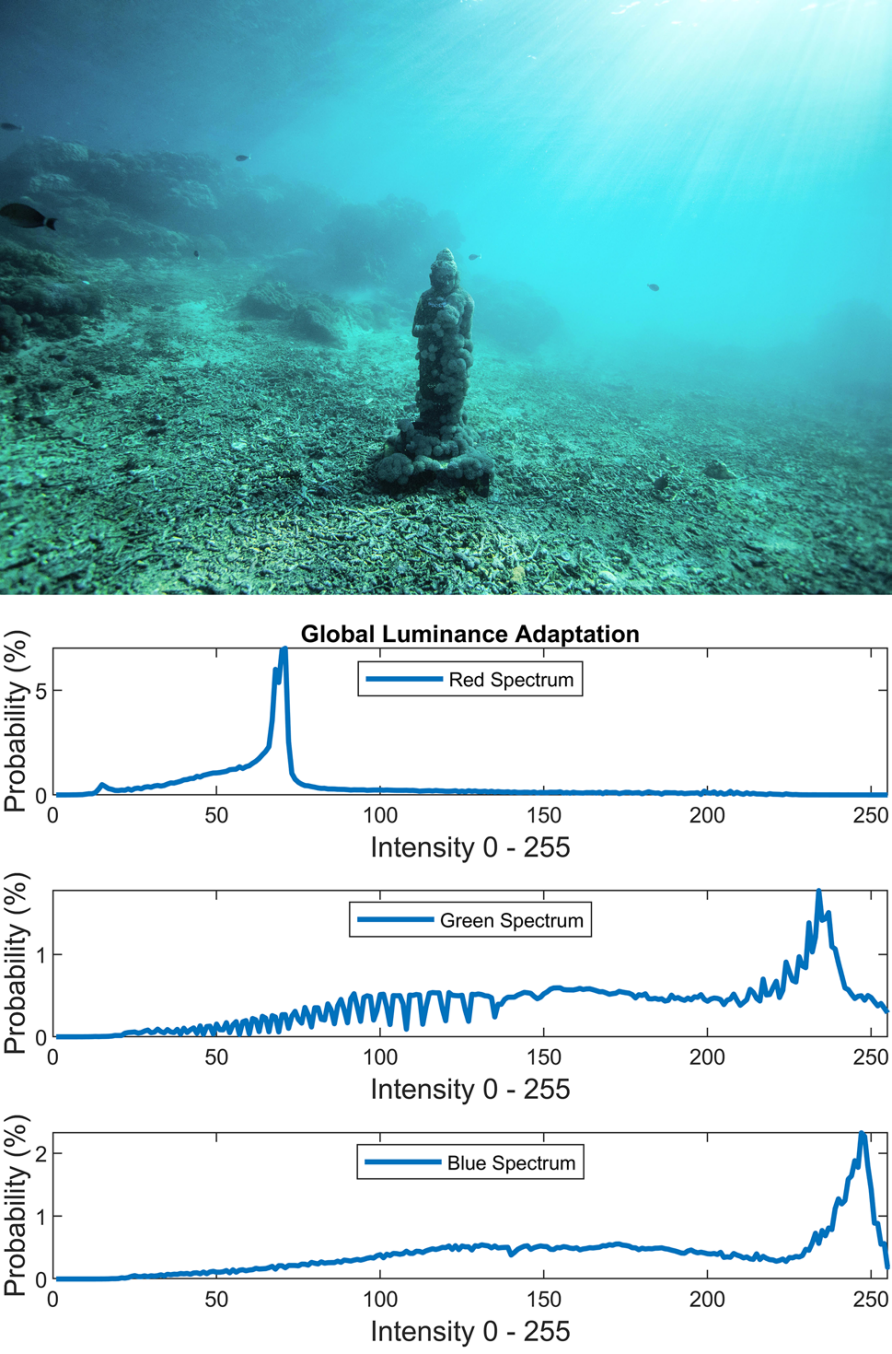
Global luminance adaptation.

**Figure 12:**
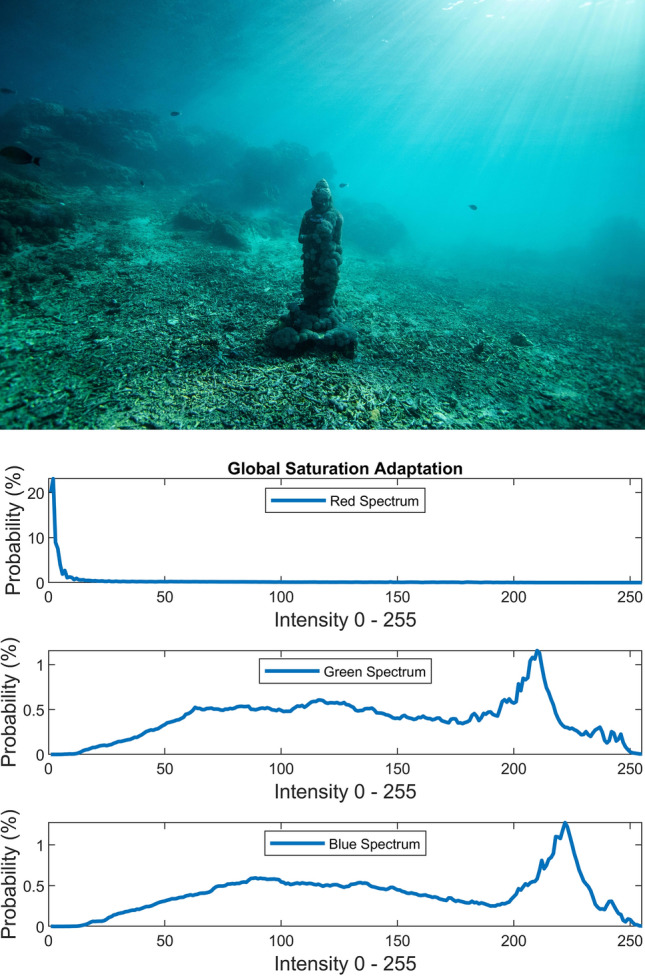
Global saturation adaptation.
- Adaptive Gamma correction is performed using the method proposed in^30^ which provides an automatic way to adaptively compute the Gamma for a given image instead of using a constant scalar. This method is computed through equations below for a gray scale image *I*:
  23 $$\gamma = \exp \left( {\frac{{1 - \left( {\mu + \sigma } \right)}}{2}} \right)$$
  24 $$c = \frac{1}{{1 + \left( {I^{\gamma } + \left( {1 - I^{\gamma } } \right)\mu^{\gamma } - 1} \right) \times heaviside\left( {0.5 - \mu + \delta } \right)}}$$
  25 $$I_{out} = cI_{in}^{\gamma }$$
  where scalar $\mu$ is the average value of the image, scalar $\sigma$ is the standard deviation of the image, and $\delta$ is an infinitesimal value to avoid zero input to Heaviside function. Figure 13 illustrates the visual and spectral effect of this method applied on Fig. 4.

**Figure 13:**
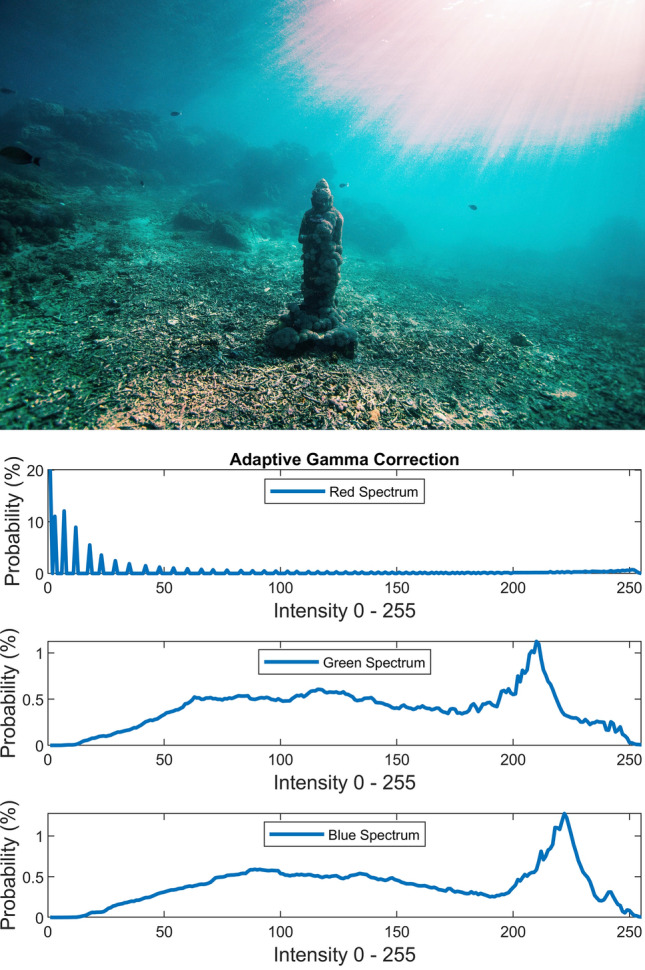
Adaptive Gamma correction.
- Red channel mean shifting and sharpening is composed of two operations. It should be noted that this approach is used in only few cases since this method requires a user defined scalar for blending. The initial operation shifts the mean of the red channel toward the gray image’s mean and then blends the green spectrum into red spectrum according to the following equation:
  26 $$I^{R} = \frac{{\mu_{Gray} }}{{\mu_{R} }} I^{R} + 0.1\left( {\mu_{G} - \mu_{R} } \right)I^{G} \left( {1 - \frac{{\mu_{Gray} }}{{\mu_{R} }}I^{R} } \right)$$
  The second operation sharpens the red channel $I^{R}$ using unsharp masking. Unsharp masking is performed by subtracting a blurred (unsharp) version of the image from the initial image. Figure 14 illustrates the visual and spectral effect of this method applied on Fig. 4.

**Figure 14:**
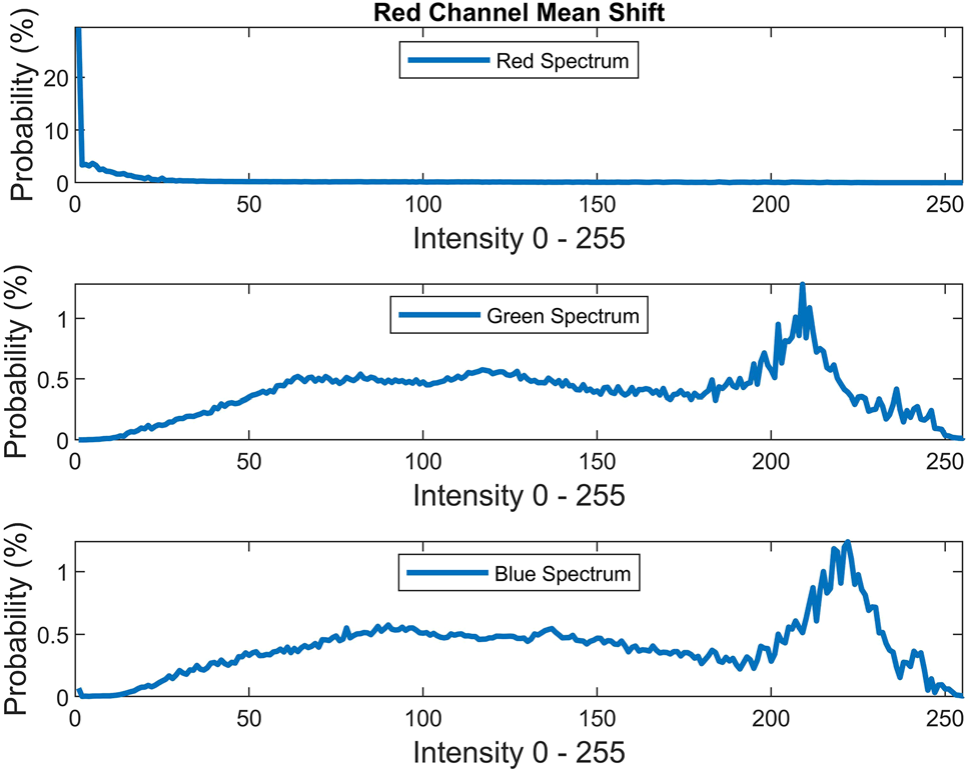
Red channel mean shifting.
- In this work, we propose the idea of even to odd middle intensity transference using look-up table to augment the probability of middle intensities (roughly doubling). After transferring the even middle intensities to their adjacent odd intensities, every odd middle intensity will roughly have a doubled probability. This phenomenon is due to the continuity of the cumulative distribution (cdf) of the discrete and continuous data. In other words, two adjacent intensities have quite slight difference in their probabilities. The selected middle intensities are between [8 and 250]. Other intensities are left intact. This method partially affects the image and has no extensive positive or negative effect on the image. Figure 15 illustrates the visual and spectral effect of this method applied on Fig. 4.

**Figure 15:**
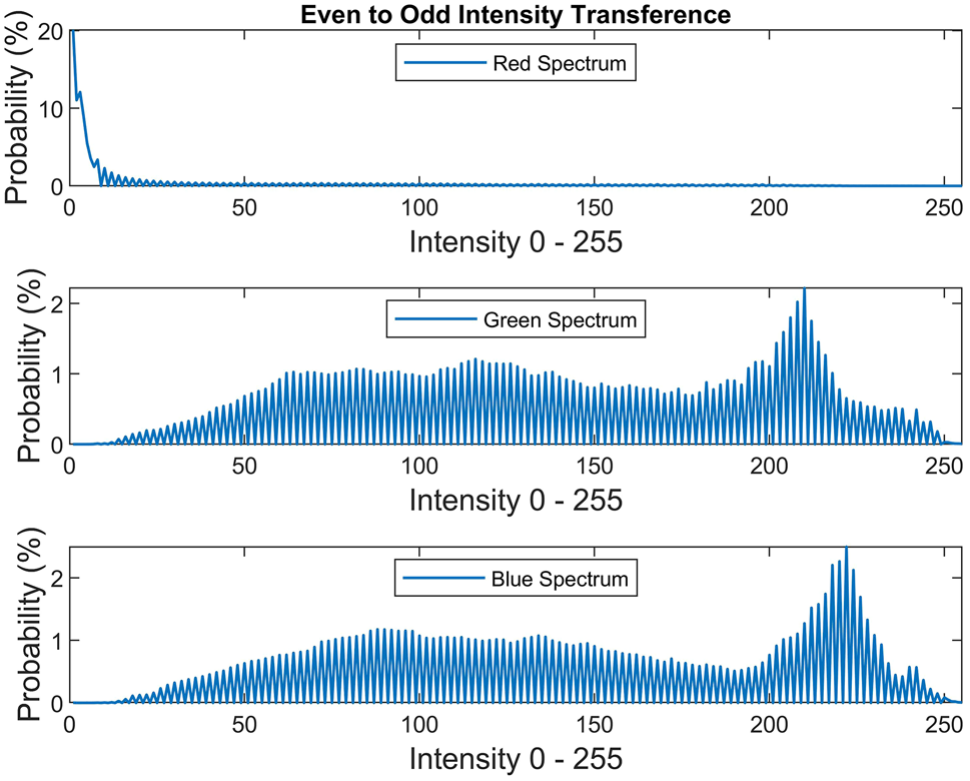
Even to odd transference.
- Green to red spectrum transference using histogram equalization is performed since we have observed many positive effects using green spectrum in contrast to blue spectrum. The transference is performed with the following equation:
  27 $$\rho_{red} = \rho_{green}$$
  where $\rho_{green}$ is the green spectrum which is used in histogram equalization. Figure 16 illustrates the visual and spectral effect of this method applied on Fig. 4.

**Figure 16:**
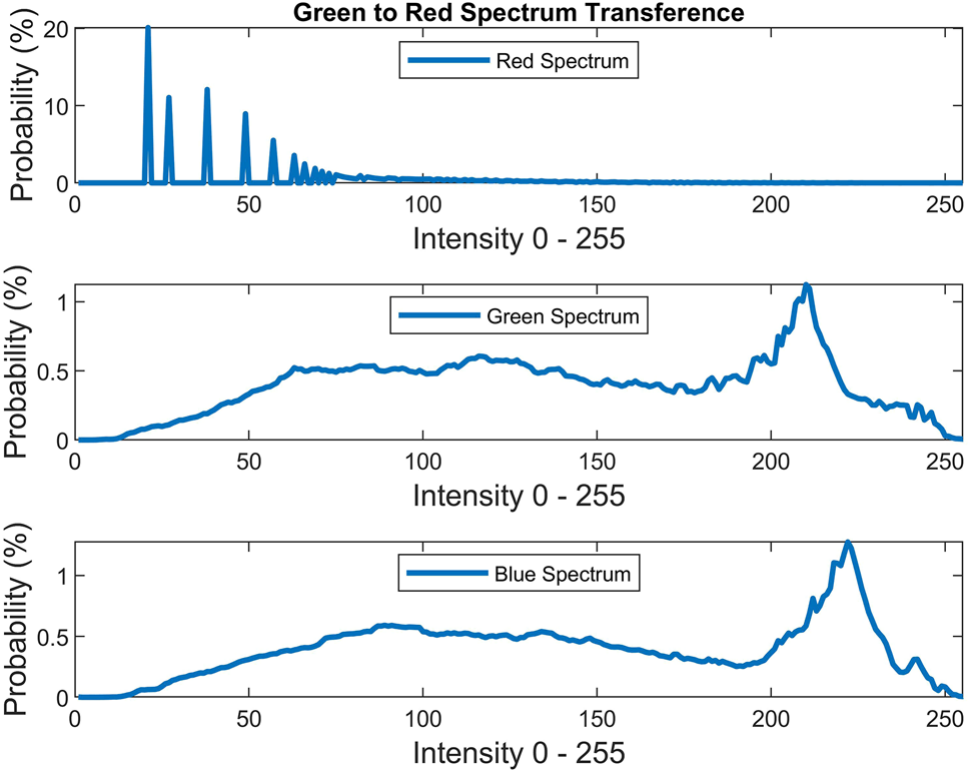
Green to red spectrum transference.
- The local brightening^31^ is used as one of our color correction methods but it should be noted that local brightening method quite degrades the overall underwater images. Due to this effect, we have set the blending option of the brightening method to (0.1).

We have used the Bootstrap Aggregation (Bagging) and Random Forests^32,33^ to create three trained models with which the three labels are generated. The first, second and third labels are generated separately through their designated trained model and then these labels are sequentially exerted on the input according to their order.

## Experimental results

A comprehensive and fair experiment of several traditional underwater image enhancement methods is performed in UIEB dataset paper^34^ from which our raw and reference images are drawn. Likewise, a comprehensive evaluation of several recent learned models (deep learning) is performed in^35^ from which we quote them in Table 1. The UIEB dataset^34^ has 890 real-world paired images (raw and reference) plus 60 challenging raw images. The images are 8-bit and taken in different situations and viewing angles and have different dimensions. The paired images facilitate error measuring in different forms such as Mean Square Error (MSE), Peak Signal to Noise Ratio (PSNR) and Structural Similarity Index for Measuring image quality (SSIM). In UIEB dataset, the degraded images are paired with the references by asking 50 volunteers to select one image out of 12 enhanced results. The enhanced results were produced by 12 traditional methods while one of the was Dive + app (with predefined settings).

**Table 1:** Full-reference image quality evaluation.

Method	MSE ↓	PSNR(dB) ↑	SSIM ↑
Original	1768.9	17.36	0.6168
Fusion-based^10^	867.9	18.7461	0.8162
Two-step-based^36^	1114.6	17.6596	0.7199
Retinex-based^37^	1353.1	16.8757	0.6233
UDCP^2^	5130.0	11.0296	0.4999
Regression-based^38^	1136.5	17.5751	0.6543
GDCP^39^	3634.5	12.5264	0.5503
Red Channel^6^	2107.3	14.8935	0.5973
Histogram prior^40^	1628.2	16.0137	0.5888
Blurriness-based^41^	1582.6	16.1371	0.6582
MCycleGAN^21^	1132.2	18.33	0.6138
URCNN^18^	2195.8	15.94	0.5972
UWGAN^42^	1853.7	16.06	0.2945
DUIENet^34^	1012.2	19.29	0.8093
DenseGAN^16^	1363.6	17.56	0.4239
UWCNN type-I^15^	2345.0	15.00	0.5306
UWCNN type-III^15^	2920.2	14.24	0.4945
Dive +	535.8	20.8408	0.8705
Our(^10^ + Haar)	745.3	22.1079	0.8105
Our(ETCA)	210.4	34.9780	0.8956

Table 1 quotes the performance of our methods as well as the performance of other methods according to^34,35^.

Our simulations are executed via Matlab software and the source is freely available for evaluation.

The speed performance depends on the image size, methods applied on the image and the processor. The larger the image, the slower the result is produced since all of our color correction algorithms are applied on the whole image. Therefore, the number of pixels has direct influence on the speed performance. The maximum latency happens when both multi-scale fusion^10^ and our proposed Haar wavelet restoration go along with each other inside the ensemble of triple algorithms. The frame rate (FPS) for red channel mean shift, contrast stretching, luminance stretching, saturation stretching, global RGB adaptation, global luminance adaptation, global saturation adaptation, DCP, even to odd transference, local brightening, green to red transference, multi-scale fusion^10^, and Haar wavelet is 0.60, 35, 0.70, 4.95, 3.08, 0.67, 3.87, 0.28, 312.0, 2.70, 5.5,0.24, and 0.93 respectively which is the average measured with an Intel Core-i3 2100–3.1 GHz processor on a 1120 × 1380 image after 300 repetitions.

The number of levels used in decomposing the input images and weights in both multi-scale fusion and Haar wavelet transform is equal to *l* = *5*. The maximum boundary of each quadrant depends on the number of histogram bins. We have used 256-bin histogram. Therefore, the maximum boundaries ${\Omega }_{1} ,{\Omega }_{2} ,{\Omega }_{3} ,{\Omega }_{4}$ will be {63,127,191,255}.

The underwater image restoration using Haar wavelet coefficients refinement has shown a superior improvement in terms of average MSE, PSNR and SSIM on the whole dataset over traditional and deep learning methods as you can see in Table1. The only superior method over our proposed traditional method is the commercial app called Dive + . As mentioned before, the images are resized to 640 × 640 in image restoration using Haar wavelet transform. Due to this, the accuracy decreases while the processing speed increases. It is possible to increase the input image to higher dimensions to increase the precision since the Gaussian pyramid of the saliency weights will carry more information to be used in the modification of the final pixel.

Our method ETCA is flexible and it is possible to use other color correction algorithms that are more robust instead of some of our current fifteen methods. As it can be seen in Table 1, the best performance among all the presented methods belongs to our proposed ETCA. Our evaluations and calculations shown that the ETCA has an average percent of MSE improvement of nearly %83.4 which is a high percentage. This means that most of the MSEs are low and only few cases are least improved.

During optimization, more than 1460 triple permutations are evaluated to reach the best ensemble of labels. The number of images on which the ETCA has applied null operation is 11.

**Figure 17:**
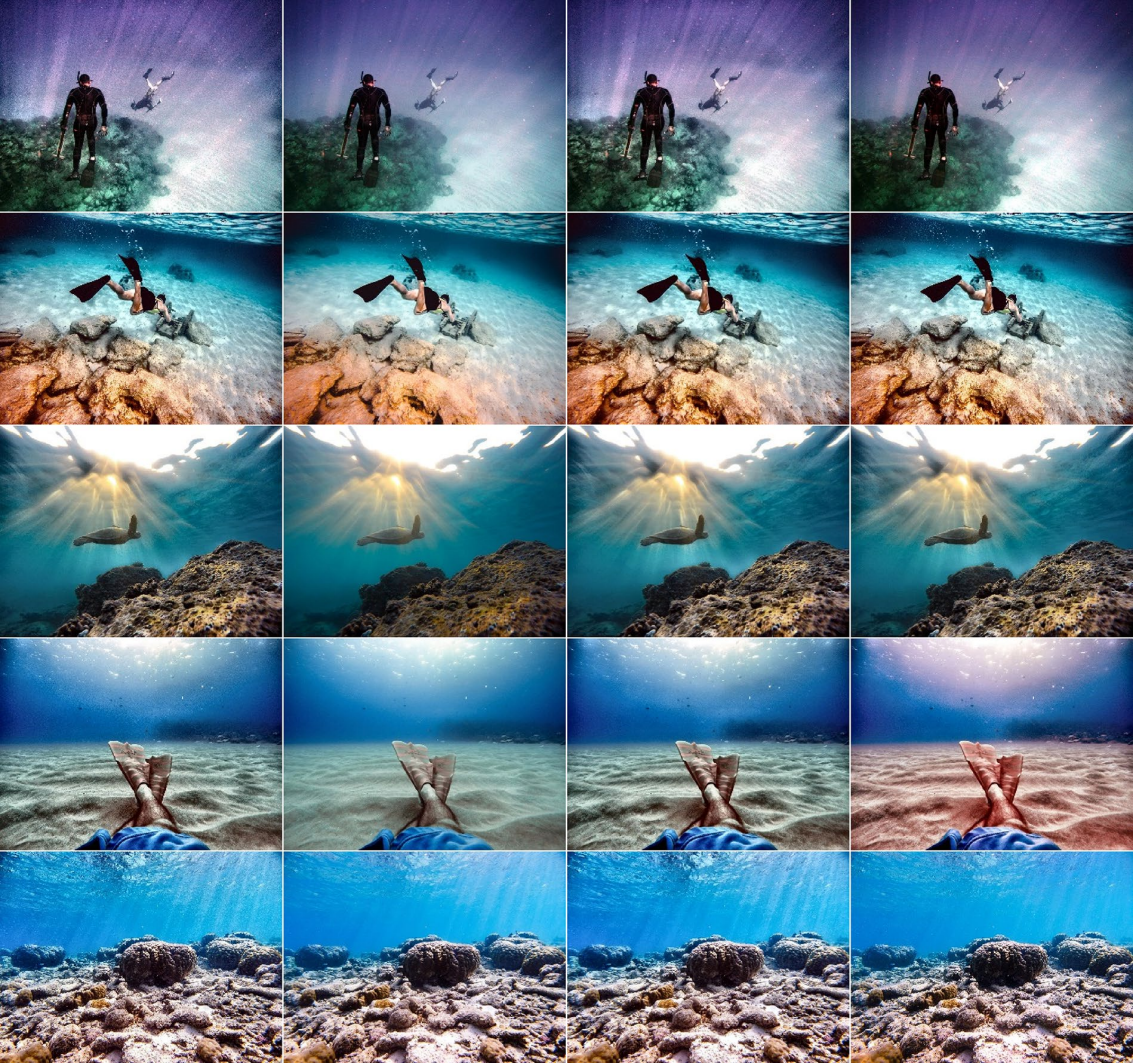
Evaluation of four methods on test images from unsplash.com: multi-scale Fusion^10^, our Haar wavelet, multi-scale Fusion^10^ plus our Haar wavelet, and proposed ETCA (from left to right).

Our technique shown limitations when the optimization process has produced weak ensemble of correction algorithms in few cases. By evaluating the restored images visually, we found that most of the UIEB dataset images are well detected and restored by histogram quadrants and only in very few cases some reddish artifacts are produced. In Fig. 17 evaluation of multi-scale fusion^10^ and our proposed methods on some test images that are not from UIEB dataset is shown (from unsplash.com).

Out of fifteen color correction algorithms, only two of them rely quite a lot on experimental user defined parameters. These two algorithm can create extreme changes when applied with inappropriate parameters. These methods include red channel mean shifting and local brightening. Both of them require a parameter for blending amount to which we have set a scalar value of (0.1).

### Conclusion

The ensemble of triple correction algorithms proves the advantageous of superposition effect of different robust and weak approaches. Our evaluations on robustness of histogram’s quadrants have proven the veracity of these features to be discriminative enough for raw images (before any correction). This indicates that with each stage of correction, the histogram disguises into a shape that causes the histogram quadrants become more indistinguishable. This situation entails a considerable risk if images are required to be characterized before the second stage. To confront this situation, we opt histogram octants instead of quadrants. Another feature of the ETCA is that it relies on few user-defined parameters.

The alternative approach to random forests and bootstrap aggregation is the pattern recognition neural networks. We have built the ETCA using pattern recognition neural networks with under hundred neurons in a network with a single hidden layer. While the quadrants are learned easily to characterize raw images, it is strikingly not feasible for characterizing a largely corrected image.

The Haar wavelet coefficient blending is our tentative plan to incorporate a dynamic contrast stretching method through blending the coefficients of a %0.5 contrast stretched image with the raw image (as a counterpart to static %1 stretching).

The idea of merging multi-scale fusion with wavelet coefficient refinement has shown a mediocre performance since the method generally produces stretched/equalized spectrums that is insufficient for all types of degradations. Nevertheless, among all the state of the art methods in Table 1, Dive + app merely outperforms ours.

The numerical and visual inspections via carrying out the global RGB adaptation, global luminance adaptation and global saturation adaptation on the raw images have shown to cause displeasing yields as an initial color correction operation for underwater images.

The global RGB adaptation exerted as the second or the third correction operation yields an image spectrum with confined upward compression based on the spectrum’s average provided that the first color correction operation broadens the initial spectrum. Likewise, the global saturation adaptation and global luminance adaptation exerted as the second or the third operation yield upward compressed saturation and luminance spectrums respectively which will dynamically amplify the saturation and luminance spectrums according to the log-average value.

The contrast stretching, luminance stretching and saturation stretching are by orders of magnitude more engrossing than three global adaptations as initial color correction methods since most of the underwater images suffer initially from a dense narrow red spectrum. These three stretching methods also to some extent, overwhelm the three global adaptation methods as the second or the third correction methods.

The idea of static %1 contrast stretching is prone to produce reddish artifacts or sometimes supplant the green water pixels with blue water pixels. This confinement brings up the idea of dynamic contrast stretching such as with variable stretching tolerance.

Likewise, the ample green and blue pixels in a scene may occasionally turn the luminance stretched image into a more displeasing greenish or bluish scene if it is applied as the initial color correction method. On the other hand, luminance stretching mostly confronts the red spectrum with an indispensable zenith (sufficiently large bin but not the peak) at zero while one or two zeniths may spawn at each end of green and/or blue spectrums (introducing wide range of pixel exposures) provided that it is not applied as the first color correction operation.

Saturation stretching confronts the red spectrum’s first quadrant (Q1) whether supplanted with an indispensable non-vain Q1 or turned down to the lower values due to imbalance between RGB spectrums, therefore, confining its usage as an initial color correction.

Saturation stretching is also prone to introduce darker colors or turn the blue underwater sunlight into bright white sunlight when it is applied as the second or third operation.

Adaptive Gamma correction supplants the initial repressed red spectrum (having large Q1 cdf and low mean) with a striking wider and turned up spectrum whose peak has been settled between Q2 and Q3. This method may occasionally create displeasing reddish artifacts.

The veracity of green and red spectrums’ resemblance is frequently seen in different environments and scenes by visual inspections of image histogram. Therefore, an engrossing tentative solution for red channel compensation is to convey the green spectrum’s distribution to red channel.

## Data Availability

The presented work is open access and is available at: https://github.com/vahidr213/Underwater-Image-Restoration-And-Enhancement-Collection/18.00**.** The UIEB dataset is downloadable from: https://li-chongyi.github.io/proj_benchmark.html.
