# Author Correction: Underwater image restoration with Haar wavelet transform and ensemble of triple correction algorithms using Bootstrap aggregation and random forests

**DOI:** 10.1038/s41598-022-19311-4

**Published:** 2022-09-07

**Authors:** Vahid Rowghanian

**Affiliations:** Independent Author, Ahvaz, Iran

Correction to: *Scientific Reports* 10.1038/s41598-022-11422-2, published online 27 May 2022

In the original version of this Article, Figures 14, 15 and 16 were incorrect. The original Figures 14, 15 and 16 and accompanying legends appear below.

**Figure 14:**
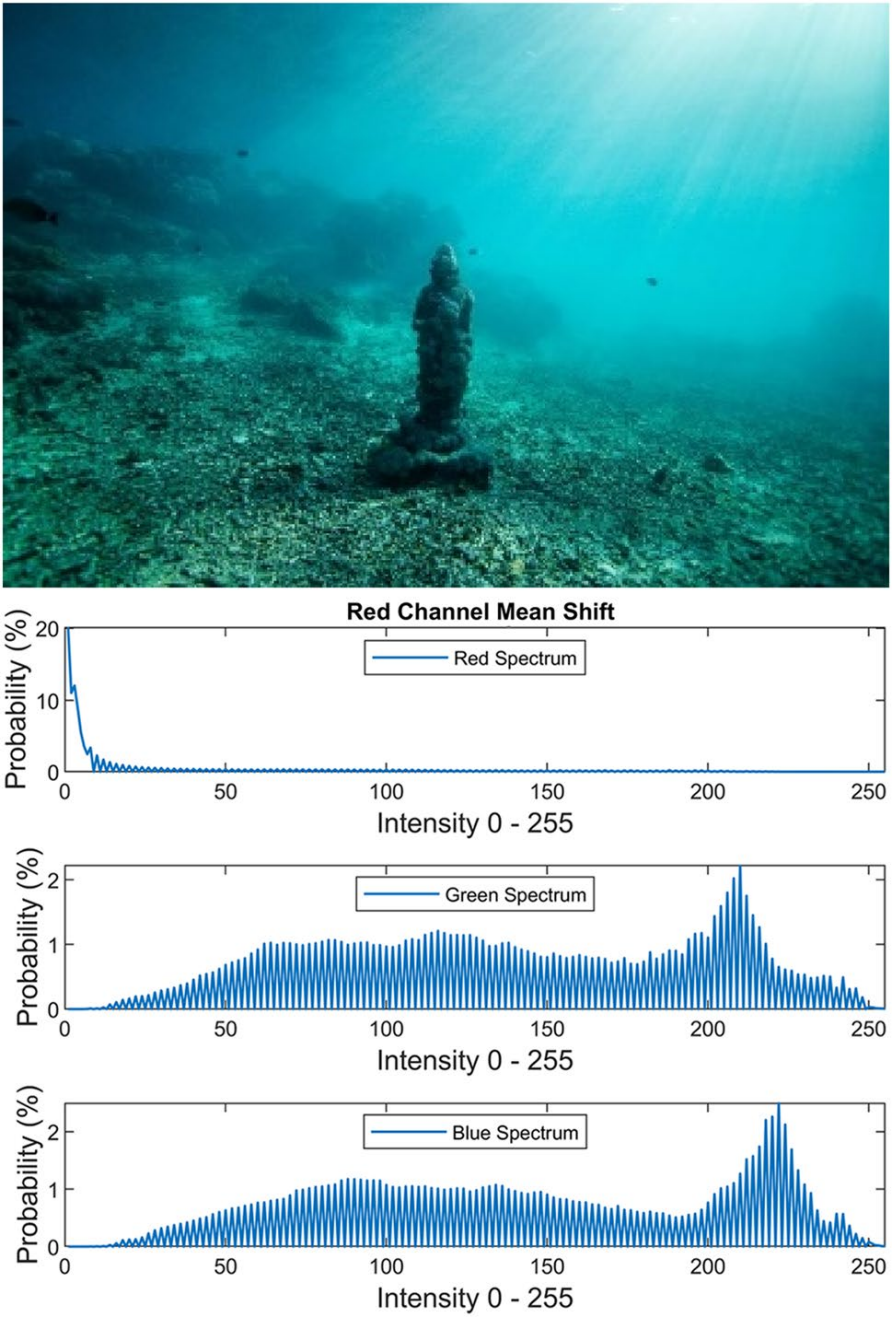
Red channel mean shifting.

**Figure 15:**
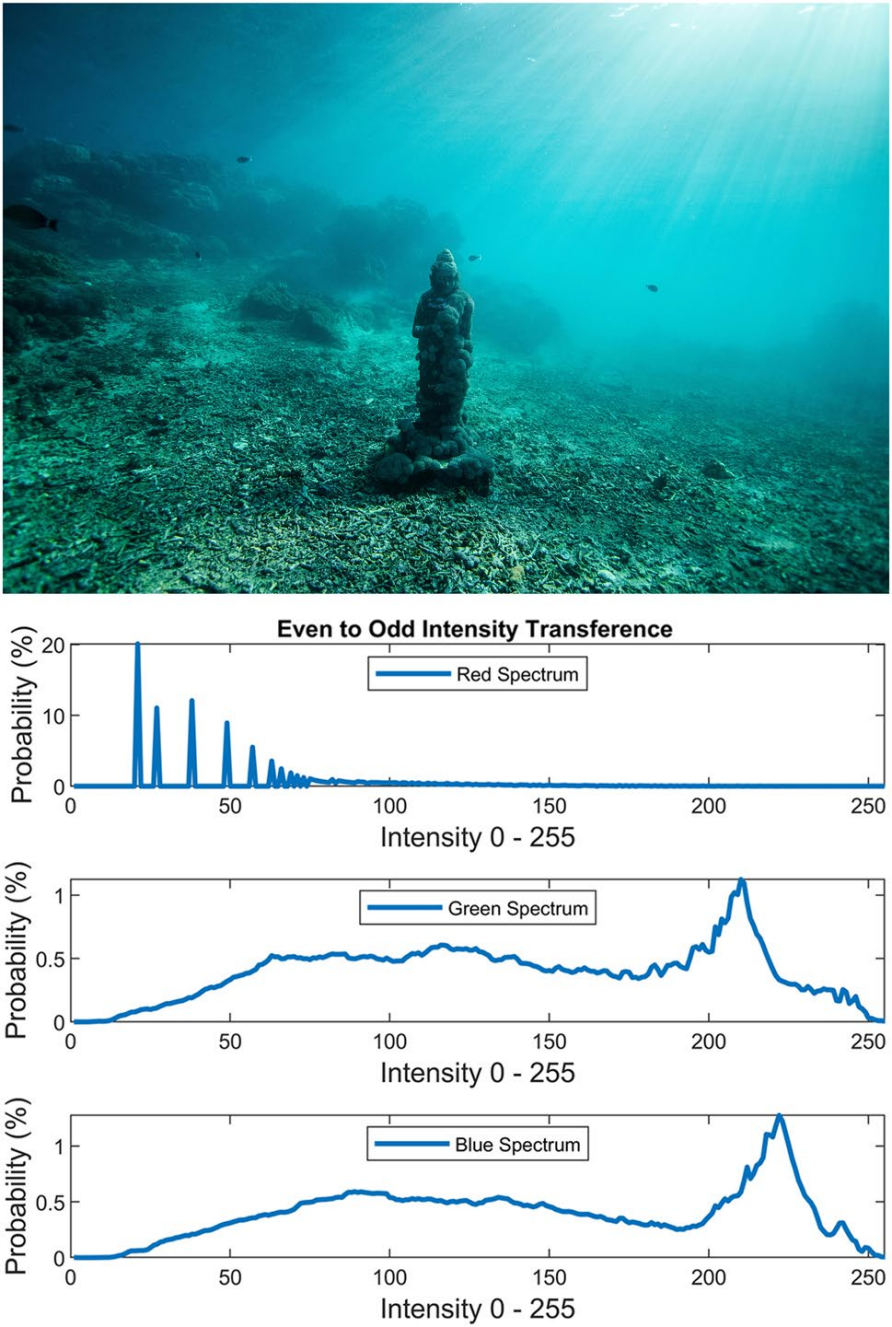
Even to odd transference.

**Figure 16:**
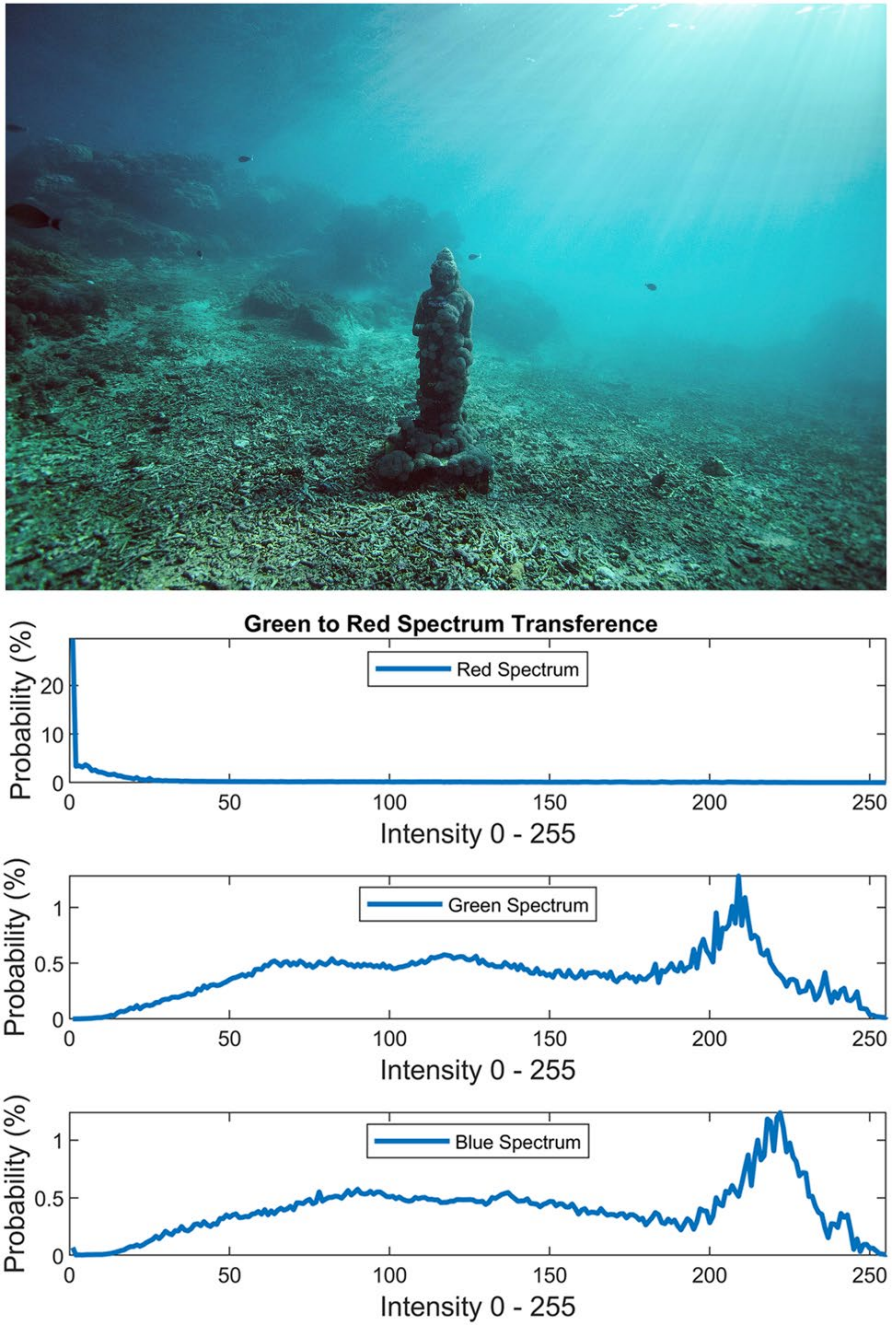
Green to red spectrum transference.

The original Article has been corrected.

